# Adaptive protein evolution through length variation of short tandem repeats in *Arabidopsis*

**DOI:** 10.1126/sciadv.add6960

**Published:** 2023-03-22

**Authors:** William B. Reinar, Anne Greulich, Ida M. Stø, Jonfinn B. Knutsen, Trond Reitan, Ole K. Tørresen, Sissel Jentoft, Melinka A. Butenko, Kjetill S. Jakobsen

**Affiliations:** ^1^ Section for Genetics and Evolutionary Biology, Department of Biosciences, University of Oslo, 0316 Oslo, Norway.; ^2^ Centre for Ecological and Evolutionary Synthesis (CEES), Department of Biosciences, University of Oslo, 0316 Oslo, Norway.

## Abstract

Intrinsically disordered protein regions are of high importance for biotic and abiotic stress responses in plants. Tracts of identical amino acids accumulate in these regions and can vary in length over generations because of expansions and retractions of short tandem repeats at the genomic level. However, little attention has been paid to what extent length variation is shaped by natural selection. By environmental association analysis on 2514 length variable tracts in 770 whole-genome sequenced *Arabidopsis thaliana*, we show that length variation in glutamine and asparagine amino acid homopolymers, as well as in interaction hotspots, correlate with local bioclimatic habitat. We determined experimentally that the promoter activity of a light-stress gene depended on polyglutamine length variants in a disordered transcription factor. Our results show that length variations affect protein function and are likely adaptive. Length variants modulating protein function at a global genomic scale has implications for understanding protein evolution and eco-evolutionary biology.

## Teaser

Length-variable amino acid tracts in plant proteins affect interactions and correlate with bio-climate.

## INTRODUCTION

Hypervariable short tandem repeats (STRs), also termed microsatellites, are present within gene transcripts and intergenic regions throughout the Tree of Life (*1*). Within the coding part of genes, STRs with a unit size of three encode repeated amino acids (homopolymers) of different lengths without generating codon frameshifts. A substantial percentage of vertebrate and plant proteins (8 to 9%) contain homopolymer tracts encoded by STRs (*2*). Unlike the average protein sequence, homopolymer evolution is driven by a different mode of mutation: DNA replication slippage. DNA replication slippage leads to altered homopolymer lengths over generations. It is established that such length variation may cause disease in humans (*3*). However, little is known regarding the effects of benign homopolymer length variation on protein function and structure and whether length variants relate to environmental adaptation.

Homopolymer tracts tend to accumulate in protein regions lacking a stable structure, termed intrinsically disordered regions (IDRs) (*4*). IDRs may, given environmental cues, facilitate a change in protein solubility that leads to specific cellular responses. Recent studies demonstrated the importance of IDRs at the phenotypic level in *Arabidopsis thaliana* (henceforth *Arabidopsis*), in relation to temperature sensing, osmotic stress sensing, and the plant immune response (*5*–*7*). Furthermore, homopolymer length variation among *Arabidopsis* accessions (i.e., *Arabidopsis* gathered from different locations) has been shown (*8*, *9*).

Here, we address whether the generation of homopolymer length variants (hypervariability) provides an important yet unexplored mode of protein evolution in plants and other organisms. We use the 1001 Genomes Consortium dataset (*10*) of whole-genome sequenced *Arabidopsis* to estimate the length of every homopolymer tract in 770 accessions collected from their local Eurasian range, which cover a wide geographical and environmental spectrum. We combine environmental association analysis with IDR protein structure predictions to investigate whether natural selection may have shaped homopolymer lengths and, furthermore, experimentally uncover a role for length variable polyglutamine (poly-Q) tracts in the activity of a transcription factor.

## RESULTS

### Coding STRs predominantly encode structurally disordered homopolymers

**Fig. 1.**
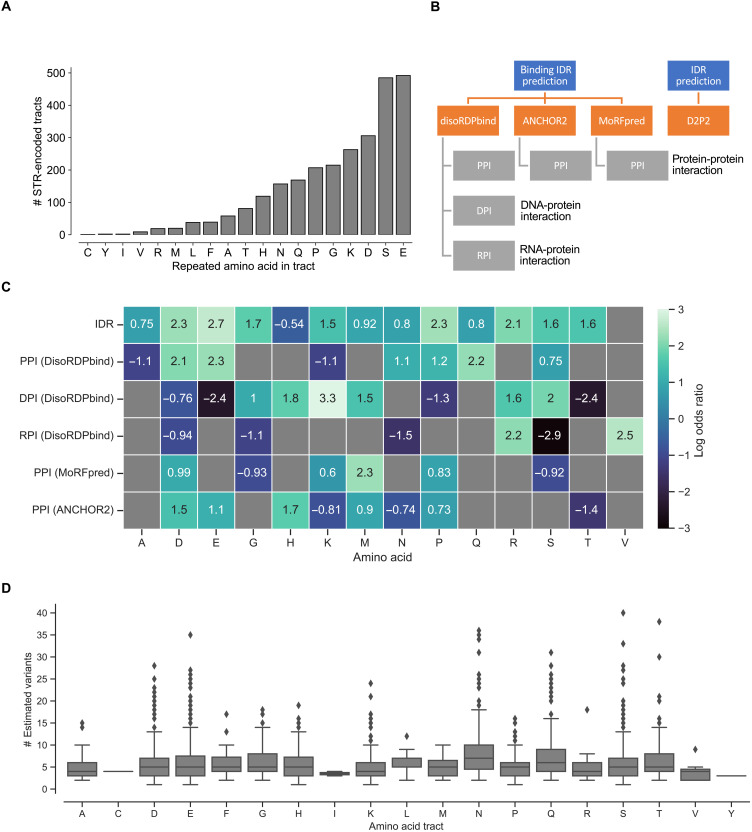
Protein coding STRs often encode disordered regions in *A. thaliana* proteins. (**A**) The bar chart shows the number of STR-encoded homopolymer tracts per type of amino acid in the *A. thaliana* reference (TAIR10) protein sequences (table S1). (**B**) Outline of prediction tools used for IDRs and disordered interaction regions. (**C**) Degree of over- or underrepresentation (two-sided Fisher’s exact test) of homopolymer tracts encoded by reference genome STRs within the predicted (IDR, PPI, DPI, and RPI) regions. Box color shading, from black to light green, is scaled with log odds ratio values. All results shown were statistically significant after Bonferroni correction for multiple testing (dataset S1). (**D**) The box plots show distributions of the estimated number of coding STR alleles in 770 different *A. thaliana* accessions (dataset S2). Amino acid legend: A, alanine; C, cysteine; D, aspartate; E, glutamate; F. phenylalanine; G, glycine; H, histidine; I, isoleucine; K, lysine; L: leucine, M, methionine; N, asparagine; P, proline; Q, glutamine; R, arginine; S, serine; T, threonine; V, valine; Y, tyrosine. Note that no STR-encoded tracts of tryptophane (W) were detected in the TAIR10 reference proteins.

We analyzed all protein coding DNA sequences in The *Arabidopsis* Information Resource 10 (TAIR10) reference genome based on the Col-0 accession (CS76778) and found the most common STR-encoded homopolymers to be polyglutamate (poly-E), polyserine (poly-S), and polyaspartate (poly-D) (Fig. 1A and table S1). To address the hypothesis that length variation in STRs could tune intermolecular interactions by introducing structural changes in disordered protein stretches, we assessed whether homopolymers in *Arabidopsis* proteins coincided with predicted IDRs, as well as with predicted disordered protein-protein, DNA-protein, and RNA-protein interaction regions (PPIs, DPIs and RPIs, respectively). The analysis scheme is depicted in Fig. 1B. To assess the extent of over- or underrepresentation of the homopolymers in IDRs and/or in disordered interaction regions, we used two-sided Fisher’s exact tests. The results are shown in Fig. 1C and dataset S1. We found that 12 of 20 different amino acid homopolymer tracts were overrepresented in IDRs, broadly mirroring patterns identified in mammalian and avian proteins (*4*). The strongest enrichments in PPI regions, predicted by DisoRDPbind were poly-E (log odds ratio, 2.3), poly-Q (log odds ratio, 2.2), and poly-D tracts (log odds ratio, 2.1). This differed from the strongest enrichments in Molecular Recognition Feature predictor (MoRFpred) regions, PPI regions (poly-M; log odds ratio, 2.3), and ANCHOR2 PPI sites (poly-H; log odds ratio, 1.7). Poly-K tracts were strongly enriched in DPI regions (log odds ratio, 3.3), and in RPI regions, the strongest enrichment was for poly-R tracts (log odds ratio, 2.2). These results show that STR-encoded homopolymers are nonrandomly distributed with regard to IDRs and disordered interaction regions. We previously genotyped STRs in the *Arabidopsis* accessions released by the 1001 Genomes Consortium and defined 770 accessions as a high-quality subset (*9*). We used this subset (dataset S2) to further explore the characteristics of length variable coding STRs. Notably, we found that polar (S, T, N, and Q) and negatively charged (D and E) homopolymers had a high number of estimated STR length variants present in the population (Fig. 1D). To better grasp the biological relevance of proteins with length variable homopolymers in IDRs and/or in disordered interaction regions, we performed network and Gene Ontology (GO) analysis of these proteins via the STRING database (*11*). The network and GO analyses showed that the protein set (1850 proteins) was more biologically connected (including physical interactions, co-mentioning in PubMed abstracts, and coexpression) than what was expected in the *Arabidopsis* background (5995 edges in the network compared to 4252 expected edges; *P* < 1.0 × 10^−16^; dataset S3). The top enriched biological process GO terms included heterochronic regulation of development (6 proteins), circadian rhythm (20 proteins), regulation of salt stress responses (12 proteins), and response to chitin (21 proteins). Notably, we found a high number of teosinte branched1, cycloidea, and proliferating cell factors 1 and 2 (TCP) family proteins (TCP2, TCP3, TCP4, TCP7, TCP10, TCP13, TCP14, TCP15, and TCP24) in the network [9 of the 24 TCP members, protein family (PFAM) enrichment *P* = 0.01; dataset S3]. TCP proteins are known to contain IDRs and to be responsive to environmental stimuli (*12*, *13*). Next, on the basis of gene expression analyses from previous work (*9*), we investigated whether length-variable STRs encoding homopolymers in IDRs or disordered interaction regions were more frequently associated with the expression of the gene that it resides in compared to other coding STRs. Supporting that DNA binding homopolymer tracts could function as autoregulatory gene expression modulators, we found that homopolymers likely to bind DNA (i.e., in DPI regions) were significantly overrepresented (fig. S1 and dataset S4). Furthermore, the tools predicted elevated protein-protein binding propensity in the poly-Q tract of the thermosensor region in early flowering 3 (ELF3), as well as in the poly-E tract of alfin-like 6 (AL6), where we previously demonstrated an effect on PPIs (*9*). This supports that the prediction tools can point to known biologically interesting regions. PPI, DPI, and RPI predictions for ELF3, AL6, and other example proteins are shown in fig. S2.

### Early-light induced protein 1 promoter activation depends on the length of poly-Q tracts in TCP14

**Fig. 2.**
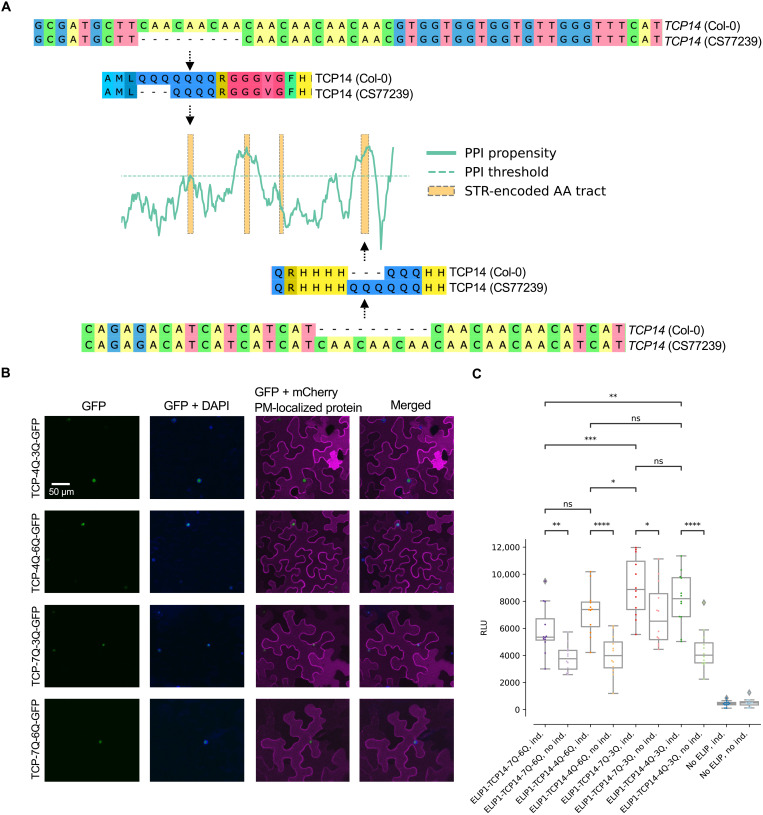
Natural allelic variation in two STR-encoded poly-Q tracts in TCP14 influences the activation of the *ELIP1* promoter. (**A**) The alignments show excerpts of the *TCP14* (Col-0) and *TCP14* (CS77239) gene and protein sequence, highlighting the two poly-Q tracts with natural allelic length variation. The cartoon shows the location of the poly-Q tracts relative to the predicted protein-protein binding propensity of the TCP14. (**B**) Transient expression of synthesized TCP14 (TCP14-4Q-3Q-GFP), TCP14 from accession CS77239 (TCP14-4Q-6Q-GFP), TCP14 from Col-0 (TCP14-7Q-3Q-GFP), and synthesized TCP14 (TCP14-7Q-6Q-GFP) in *N. benthamiana* leaves. The images show that TCP14-4Q-3Q-GFP, TCP14-4Q-6Q-GFP, TCP14-7Q-3Q-GFP, and TCP14-7Q-6Q-GFP localize to the nuclei. A protein known to localize to the plasma membrane (PM) was used to outline the PM of the cells. (**C**) The luciferase output (RLU) of the *ELIP1* promoter is dependent on the Q tracts in TCP14. The differences in luciferase output show that all TCP14 variants activate the *ELIP1* promoter and that when both tracts are changed so is the strength of the activation. The asterisks indicate the result of Welch *t* test [****P < 0.0001, ***P < 0.001, **P < 0.01, and *P < 0.05; not significant (ns), *P* > 0.05]. *N* = 12 (for technical replicates, see dataset S14). Ind., induction.

**Fig. 3.**
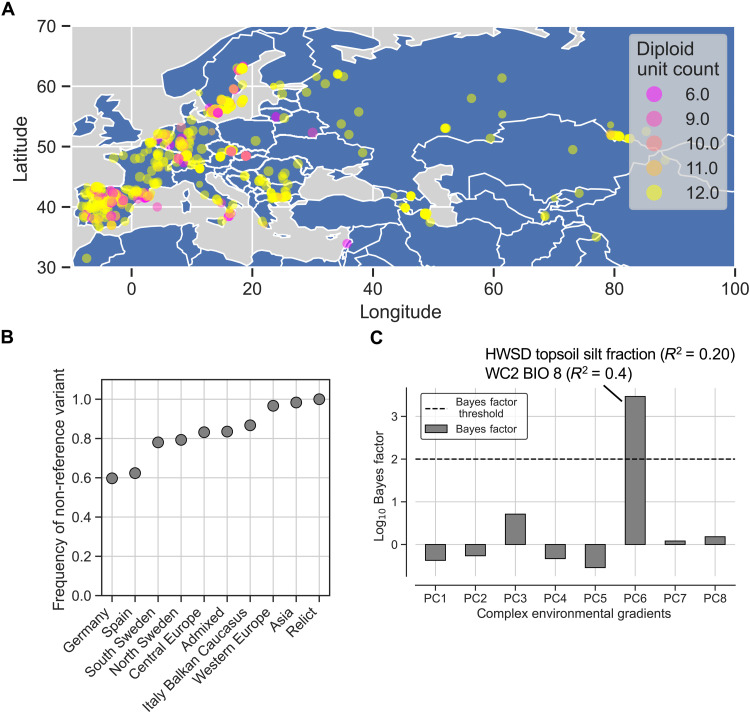
Poly-Q tract length variation in TCP14 associates with temperature and edaphic variables. (**A**) Map of the Eurasian *A.. thaliana* accessions colored by the estimated diploid unit count of the second poly-Q tract in the TCP14 protein, including heterozygous variants. (**B**) The allele frequencies (*y* axis) of the nonreference variants in the 10 groups (*x* axis) designated by the 1001 Genomes Consortium (*10*). (**C**) The bar plot shows the Bayes factors (*y* axis) that resulted from Bayenv2.0 in models where the variation in allele frequencies was treated as a response to variation in seven complex environmental gradients (PC 1 to 7, *x* axis), given the average genetic distance between groups (fig. S3). The top positive and negative correlation (squared Pearson’s *R*) between the complex environmental gradient PC6 and single environmental variables are highlighted. WC2 BIO8, mean temperature of the wettest quarter.

Several members of the TCP transcription factor family had STR-encoded homopolymers predicted to be in IDRs and/or in disordered interaction regions (dataset S3). One member of this family, TCP14, activates the photoprotective early-light induced proteins (ELIPs), which are involved in plant responses to high light stress (*14*–*16*). The expression of *ELIP1* is directly regulated by the binding of TCP14 to *up1* elements in the *ELIP* promoter regions. TCP14 acts epistatic to the Dana-like zinc finger domain–containing transcriptional regulator ORANGE (OR). OR physically interacts with TCP14 in the nucleus and represses its transactivation activity, leading to reduced transcriptional levels of *ELIP1*, reduction in chlorophyll biosynthesis, and delays of thylakoid membranes in plastids of germinating cotyledons. This repression decreases upon illumination when the nuclear localization of OR is diminished and accumulation of TCP14 in the nucleus derepresses chloroplast biogenesis during hypocotyl de-etiolation by increased *ELIP1* transcription (*15*). We found TCP14 to contain poly-Q tracts that overlapped predicted disordered PPI regions (fig. S2) and that varied in length in different *Arabidopsis* accessions. We experimentally tested the functional relevance of poly-Q tracts in TCP14 by quantifying the ability of the different homopolymer length variants to activate transcription of the *ELIP1* gene. The two poly-Q tracts that we focused on overlap with predicted disordered PPI sites (Fig. 2A), and we envisioned that length variation in these poly-Q tracts would influence the ability of TCP14 to activate *ELIP1*. We tested our hypothesis using TCP14 variants from accessions that only differed in these two tracts in the TCP14 protein sequence. The Col-0 accession had seven repeated Qs in the first tract and three repeated Qs in the second tract (TCP14-7Q-3Q). The Bulgarian accession Schip-1 (CS77239) had four repeated Qs in the first tract and six repeated Qs in the second tract (TCP14-4Q-6Q). No other amino acid level differences were present between the two accessions (dataset S5). To isolate the total effects of the first and the second tract, we synthesized and experimentally tested all the remaining combinations (7Q-6Q and 4Q-3Q). To verify that the activity of the *ELIP1* promoter (*pELIP1*) was significantly altered by the length of the TCP14 poly-Q tracts, we performed a luciferase assay in *Nicotiana benthamiana* (*N. benthamiana*) leaves where the luciferase luminescence signal [relative light units (RLU)] was a determinate of promoter activity. We coinfiltrated a *pELIP1:LUC* construct together with the different TCP14 proteins coupled to green fluorescent protein (GFP) in *N. benthamiana* leaves (TCP14-Q_n_-Q_n_). An estradiol-inducible 35*S* promoter was used to drive expression of the TCP14-Q_n_-Q_n_-GFP variants to achieve comparable expression levels for the fusion proteins. TCP14-7Q-3Q-GFP, TCP14-4Q-6Q-GFP, TCP14-7Q-6Q-GFP, and TCP1-4Q-3Q-GFP localized to the nucleus in *N. benthamiana* leaf cells (Fig. 2B). As negative controls, we measured the luciferase luminescence signals in assays containing the constructs without adding estradiol, as well as assays without *pELIP1:LUC* (Fig. 2C). Our results show that a simultaneous length change in both tracts produced significantly different luciferase outputs (7Q-6Q versus 4Q-3Q: Welch *t* test *P* = 0.007 and 4Q-6Q versus 7Q-3Q: Welch *t* test *P* = 0.03) and that the last tract yielded a significant difference only when the first tract was 7Q (7Q-6Q versus 7Q-3Q, Welch *t* test *P* = 0.0008). Changes in only the first tract did not produce any significant differences (Fig. 2C). We conclude that both tracts are involved with *ELIP1* promoter activation but that the last tract is more important. These results show that natural allelic variations in the poly-Q tracts alters *ELIP1* promoter activation, and given the important role of ELIP1 in light stress toleration, this may reflect the ability of different accessions to respond to light stress. We grouped the 770 accessions by the length of the poly-Q tract (Fig. 3A) and tested whether the poly-Q tract length correlated with environmental variables retrieved from (*17*). We investigated both linear and nonlinear effects of the poly-Q tract using ordinary least squares (OLS) regression, and the top correlation of the nonlinear effect model (*R*^2^ = 0.031, *P* < 0.0001) was with global horizontal irradiation, consistent with a scenario where the poly-Q tract has a role in light-driven responses (table S2). Next, we investigated the poly-Q tract variant allele frequencies, based on the group designation inferred by the 1001 Genomes Consortium (*10*), and found that the “Relict” and “Asia” accessions were fixed for nonreference variants, while approximately 35% of the “Germany” and “Spain” accessions had the reference variant (three repeated Qs; Fig. 3B). We tested whether these differences in allele frequencies could be explained by environmental gradients by using Bayesian modeling (Bayenv 2.0), which took the genetic similarities between the groups into account (fig. S3). Given the genetic structure and the variation in mean environment, Bayenv2.0 calculated that the poly-Q variant allele frequencies could be explained by a complex environmental gradient that primarily captured variation in soil silt content and temperature (Fig. 3C).

### Environmental correlations are driven by disorder, binding propensities, and amino acid type

**Fig. 4.**
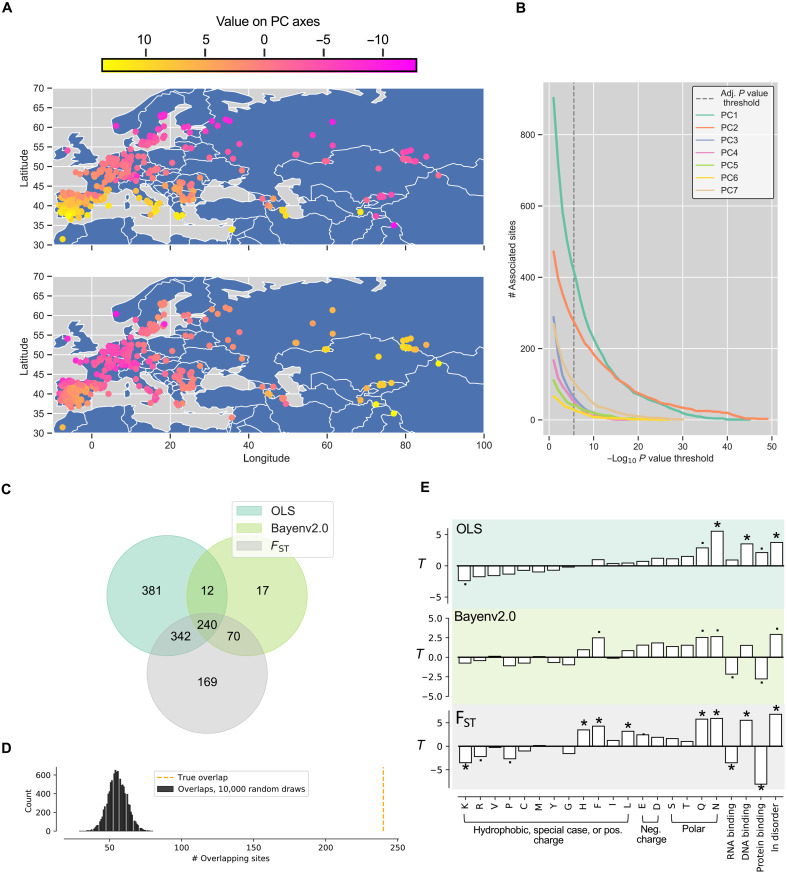
Natural allelic length variation in STRs associates with environmental gradients. (**A**) The maps show the sampling origins of the 770 *A. thaliana* accessions. Samples are colored by their position on the environmental PC axes 1 and 2 (see color bar) used in the OLS analyses. (**B**) The number of protein coding STR sites (*y* axis) associated with the environmental PC axes 1 to 7 as a function of the confidence of the environmental association (−log_10_ *P* values). (**C**) Overlap between protein coding STRs that correlated with the complex environmental gradients from the OLS environmental association analysis, the Bayenv2.0 environmental association analysis, and the pairwise *F*_ST_ analysis. (**D**) The histogram shows the distribution of the expected number of overlaps given that the candidate sites were drawn at random, without replacement. The orange, stapled line shows the number of true overlaps. (**E**) The results of multiple linear regression where variation in either the *R*^2^ (OLS, top), the Bayes factor (Bayenv 2.0, middle), or the *F*_ST_ (F_ST_ analysis) served as a response and the type of amino acid, the disorder predictions, and the binding propensity predictions served as explanatory variables. The *y* axes show the *t* score, where positive values correspond to a positive coefficient and negative values correspond to a negative coefficient in the model (dataset S13). Note that the effect of the amino acid was relative to the baseline amino acid, set to be alanine. Asterisks denote a *P* value below the Bonferroni-corrected α threshold (0.002), and dots denote *P* values below the α threshold (0.05). Amino acid legend: C, cysteine; D, aspartate; E, glutamate; F, phenylalanine; G, glycine; H, histidine; I, isoleucine; K, lysine; L, leucine; M, methionine; N, asparagine; P, proline; Q, glutamine; R, arginine; S, serine; T, threonine; V, valine; Y, tyrosine.

Next, we explored the relationship between natural allelic variation in all the protein coding STRs that we detected in the Col-0 reference genome and the environmental origin of the accessions. Eighty-eight numerical variables had complete measurements for all the 770 accessions (dataset S6). As many of these variables were highly correlated, we decomposed the information to seven principal components (PCs) that together explained 80% of the total variation (fig. S4). The top correlations between the individual environmental variables and PC axes 1 to 7 are shown in table S4, and the accessions’ values along the axes are available in dataset S7 (PCA plot shown in fig. S5). We regressed the seven environmental PC axes on our estimates of allelic variation in 2514 protein coding STRs, one site and one environmental axis at a time (dataset S8). The accessions’ original sampling location colored by their position along PC1 and PC2 are shown in Fig. 4A. As a negative control, we used mock STR genotypes (dataset S9). We separately treated STR alleles as continuous or categorical values to assess whether linear or nonlinear effects best explained the environmental variation. In 24% of tests with multiallelic STRs, nonlinear effects better explained the environmental variation compared to linear effects. For further analysis, we kept the top associations (by *P* value) per PC axis and per STR. Of the 2514 protein coding STRs, 969 had length variation best associated with PC axis 1 (Fig. 4B and dataset S8). This axis mostly represent variation in spring maximum temperatures (table S4). At the highest confidence levels (i.e., lowest *P* values), associations with PC axis 2 were more common (Fig. 4B and dataset S8). PC axis 2 best represents variation in net primary production sensitivity to precipitation and temperature seasonality (table S4). A total of 0.3% of the tests with mock genotypes produced significant associations with a PC axis after multiple test (Bonferroni) correction, compared to 9.4% of the estimated genotypes (datasets S9 and S8). Note that we cannot completely rule out that the associations are confounded with the population structuring along the environmental gradients (fig. S6). However, when controlling for the pairwise genetic similarity with putative neutral markers (fig. S7), *P* value distributions were right skewed (fig. S8), and 23 coding STRs were still significant after Bonferroni correction (dataset S10). Next, we tested whether the amino acid type and the disorder prediction state of homopolymer tracts were significant explanatory variables in multiple regression models with the outputs of OLS (*R*^2^), Bayenv2.0 (Bayes factor), and fixation index (*F*_ST_) analyses as responses (datasets S11 and S12). To run Bayenv2.0 and *F*_ST_ analyses, we converted multiallelic variants into biallelic variants using the frequency of the most common variant and the combined frequency of the less common variants per STR, per *Arabidopsis* subpopulation. We decomposed the variation in environmental means as previously, retaining all PC axes that explained more than 1% of the total variation in means, yielding eight environmental PCs (fig. S9). We found a high overlap between *F*_ST_ outliers identified by the pairwise *F*_ST_ analysis and the candidate sites that resulted from the OLS and Bayenv2.0 models (240 overlaps versus 56 mean expected overlaps; Fig. 4, C and D). For every homopolymer tract, we treated the squared correlation (*R*^2^ from OLS), the Bayes factor, or the *F*_ST_ as the response. As explanatory variables, we used the homopolymer tract type (poly-N, poly-S, etc.) and the IDR/IDR binding prediction (PPIs, DPIs, and RPIs) as explanatory variables. The three simple models only explained a small fraction of the variation (0.4 to 1.1%; dataset S13) but together agreed that disordered tracts (three of three), poly-N tracts (three of three), and poly-Q tracts (three of three) produce significantly higher *R*^2^/Bayes factor/*F*_ST_ values (Fig. 4E). These results suggest that homopolymer tracts with these features are especially interesting as candidates in further functional studies, as they may serve important adaptive roles in *Arabidopsis.*

## DISCUSSION

In this study, we performed a global analysis of protein coding STRs in *Arabidopsis* accessions to explore how coding length variation has the potential to affect protein function and how length variations relate to environmental conditions. Our protein structure analyses indicated that coding STRs promote disorder in protein structure and that STRs in disordered regions often are predicted to bind other proteins, DNA or RNA (Fig. 1C). The nonrandom distribution of STR-encoded amino acids with regard to protein structure implies that protein level length variation, caused by random replication slippage on the DNA level, predominantly affects regions of proteins predicted to be disordered. By using the wealth of knowledge resulting from decades of functional studies with *Arabidopsis* as a model plant, our protein network enrichment analysis (dataset S3) revealed that proteins encoded in part by structural disorder-promoting STRs are not a random subset of the *Arabidopsis* proteome but are heavily involved in circadian rhythms, defense responses, and responses to salt stress. Circadian rhythms are intrinsically linked to variation in daylight and temperature and plays a role in growth and development, as well as abiotic and biotic stress responses (*18*–*20*). The enrichment of circadian, biotic, and abiotic functions in proteins with disordered regions is consistent with a scenario where functional variation in these regions has been important for the expansion of *Arabidopsis* across the northern hemisphere.

The results of our experimental approaches on TCP14 support that the prediction tools pointed to functional disordered amino acid tracts within the TCP14 protein sequence (Fig. 2). The Q tracts in TCP14 were predicted to have elevated protein-protein binding propensities, making it likely that differential downstream interacting *ELIP1* promoter activation was caused by an altered PPI between TCP14 and OR, as OR is known to bind TCP14 and represses its ability to activate the *ELIP1* promoter (*15*). Further experiments on TCP14 would be necessary to fully test this hypothesis, and follow-up experiments on additional candidate tracts, similar to Jung *et al*. (*5*), will be key to understanding the extent of the phenotypic consequences of protein coding STR length variation.

Here, we have demonstrated that length variation of coding STRs is associated with various environmental adaptations in *Arabidopsis* accessions. On the basis of the knowledge from other plant, fungal, animal, and bacterial species, coding STRs are found at high frequencies throughout the Tree of Life (*1*, *2*, *21*–*27*), and there are some published examples of functional consequences of tract length variation on specific proteins. These examples include a variable glycine-threonine tract that maintains the circadian rhythm in response to temperature in *Drosophila* (*28*). In yeast, a variable poly-Q tract in a transcriptional regulator protein influenced fitness under different growth regimes (*22*). A variable poly-Q tract in combination with a variable poly-A tract facilitates swift evolution of limb and skull morphology in canids (*29*). *Arabidopsis* is, however, one of the few species where a large collection of whole-genome sequenced accessions (adapted to very different local environments) is available. This made it possible to perform a global analysis of homopolymer tract length variation and to find candidate proteins exhibiting environmentally associated length variants. In an even further and broader perspective, the nature of STR length variation somewhat resembles that of epigenetic changes (epimutations) in that both STR length variations and epimutations occur at a rapid mode and both STR variation and epimutations may be involved in local adaptation. However, the transgenerational effect of epigenetics is not completely clear, while STR mutations acting at the DNA level are inheritably stable. In turn, this is likely to provide rapid adaptation under specific (and even recurrent changing) selection regimes due to the high length mutation rates and the standing variation in the populations.

Our environmental association analyses showed that specific homopolymer tracts (poly-N and poly-Q), as well as tracts predicted to be disordered had length variation associated with complex environmental gradients (Fig. 4E). Hence, our data suggests that natural selection has driven the STR length variation between ecologically adapted populations and, consequently, the allelic variation in these tracts as a response to differences in light, temperature, temperature variability, soil content, and other more complex gradients. Furthermore, the presence of such tracts in proteins facilitates a rapid fine-tuning of protein function in plants and likely other groups of organisms because coding STRs are ubiquitous throughout the Tree of Life.

## MATERIALS AND METHODS

### Disorder and disordered binding site predictions

We downloaded *A. thaliana* reference (TAIR10) proteome disorder consensus predictions from Database of Disordered Protein Predictions (d2p2) (*30*). d2p2 itself uses nine different predictors: VLXT, VSL2b, PrDOS, PV2, IUPred-S, IUPred-L, Espritz-N, Espritz-X, and Espritz-D (*31*–*36*). d2p2 requires that 75% of tools should agree to designate disorder. From these consensus ranges, we generated Browser Extensible Data (BED) files. To find overlaps with STR-encoded amino acids, we first ran Tandem Repeats Finder with standard options (*37*) on the TAIR10 coding DNA sequences retrieved from arabidopsis.org. This allowed us to map the STR on the coding DNA transcript level to the protein sequence. Hence, we generated another BED file containing amino acids encoded by STRs. We used Pybedtools with the “intersect” command to assess overlap of STR-encoded amino acids and the predicted regions. Pybedtools is a Python wrapper of BEDTools (*38*, *39*). For predicting disordered binding regions, we used DisoRDPbind predictions, MoRFpred predictions, and ANCHOR2 predictions (*40*–*42*). For retrieving DisoRDPbind predictions, we accessed the DisoRDPbind web server (http://biomine.cs.vcu.edu/servers/DisoRDPbind/). To retrieve MoRFpred predictions, we uploaded protein sequences to the fast MoRFpred web server available at http://biomine.cs.vcu.edu/servers/fMoRFpred/ (*43*). To retrieve ANCHOR2 predictions, we ran the IUApred2A tool locally (*44*). The different tools’ output result files were parsed to generate BED files, and overlap was quantified using Pybedtools. To link the STR-encoded homopolymer coordinates back to the genomic coordinates of the STR, we matched transcript IDs from the coding DNA sequence FASTA file to the genome annotation Generic Feature Format file (from arabidopsis.org) and then located the identical motifs detected by Tandem Repeats Finder in the TAIR10 genome sequence and in the coding DNA sequence FASTA file.

### Allelic variation in STRs and neutral single-nucleotide polymorphisms

We previously scored the number of units in each (diploid) STR allele across the *Arabidopsis* accessions (*9*). An STR-specific variant caller, haplotype inference and phasing for STRs (*45*), was used to call the variants. We used the same STR unit counts in this study but for an expanded set of accessions (770), and only STRs that encoded a minimum of four repeated amino acids. We constructed the putative neutral single-nucleotide polymorphism (SNP) matrix for the 770 accessions as in our previous study (*9*). Briefly, we drew random, common (major allele frequency < 0.9) intergenic SNPs that we pruned for linkage disequilibrium using the “locate_unlinked” function of the Python package scikit.allel (parameters: size = 1000, step = 20, and threshold = 0.1) (*46*). From these SNPs, we calculated the pairwise correlation between accessions and standardized the output matrix. The second TCP14 poly-Q tract highlighted in this study was too short, i.e., only three CAA repeats in the TAIR10 (Col-0) reference genome, to be detected by the initial Tandems Repeat Finder scan. Thus, we pointed to this site directly for genotyping with bcftools “mpileup” and “call” (*47*) to investigate the natural variation in the accessions.

### Bayesian modeling with Bayenv2.0

To format our genotyping data for use with Bayenv, we used the variant calling format (VCF) file originating from the STR variant calling of the 1001 genomes, as described in (*9*). We read the VCF with the “allel.GenotypeArray” function of scikit-allel. We used the “count_alleles_subpops” function and the “to_frequencies” function to retrieve allele frequencies per subpopulation. We used the variance-covariance matrix produced by Bayenv2.0 after 100,000 iterations as the estimate of genetic structure, which was based on the allele frequencies of a linkage disequilibrium-pruned set of all coding STRs (pruning parameters: size = 10, step = 1, and threshold = 0.1). Last, we ran Bayenv2.0 with the variance-covariance matrix as parameter -m, as described in the Bayenv2.0 documentation. A log_10_ Bayes Factor larger than one is usually interpreted as strong evidence, while a BF larger than two is often interpreted as decisive evidence (*48*).

### *F*_ST_ outlier analysis

We used allele frequency data per subpopulation to calculate pairwise the Hudson’s *F*_ST_ of all coding STRs from the 10 subpopulations (45 comparisons), with the “hudson_fst” function from the scikit-allel Python package. Outliers were defined as sites with *F*_ST_ values above the global 95th percentile of the empirical distribution.

### Bioclimatic variables

We retrieved the full set of environmental variables retrieved by Ferrero-Serrano and Assmann (*17*) and dropped measurements that were not fully covered in all samples, which left 88 different environmental variables (dataset S6). First, we scaled the variables using the “sklearn.preprocessing.scale” and then the “sklearn.decomposition.pca” functions of the “sklearn” Python module (*49*) to perform the decomposition of the 88 variables. For the use of bioclimatic variables with Bayenv2.0, we decomposed the mean values per subpopulation based on all the 197 variables.

### Cloning and transient expression of proteins

*Arabidopsis* accession CS77239 was ordered from Nottingham *Arabidopsis* Stock Center. Clonal genes of TCP14-7Q-6Q and TCP14-4Q-3Q were ordered from Twist Bioscience. The DNA sequences encoding TCP14 from Col-0 (TCP14-7Q-3Q) and TCP14 (TCP14-4Q-6Q) from accession CS77239, as well as the ordered genes encoding TCP14-7Q-6Q and TCP14-4Q-3Q were cloned in frame with an expression vector containing an 35*S* estradiol-inducible promoter and a C-terminal fluorescent molecule of GFP using the Invitrogen Gateway cloning system (*50*). The *ELIP1* promoter (*pELIP1*) was defined as the region 2000–base pair upstream of the *ELIP1* start codon and amplified from Col-0 genomic DNA. *pELIP1* was cloned into the R4pGWB635 vector (*51*, *52*) containing the LUC gene, creating the *pELIP1:LUC* construct. Primers are listed in table S3. Plasmids were transformed into *Agrobacterium tumefaciens* C58 and further used for transient expression in *N. benthamiana* leaves following a previously described protocol (*53*).

### Luciferase assay

*N. benthamiana* leaves were coinfiltrated with *ELIP1:LUC* and one of the TCP14 constructs individually. The empty vector pab117 served as a no *ELIP1:LUC* negative control. Leaves were cut into discs of 3 mm in diameter and incubated in a 10 mM estradiol solution for 3 hours to induce gene expression of TCP14. Uninduced leaf discs for each construct were used as a negative control for TCP14 activation of *ELIP1:LUC*. Leaf discs were individually transferred to wells in a 96-well plate treated with d-luciferin (0.5 mg/ml) and kept in darkness for 105 min before luminescence was detected using a BioTek Synergy H1 microplate reader (Agilent). For imaging, leave discs were incubated in a 10 mM estradiol and 4′,6-diamidino-2-phenylindole solution mix (2.5 μg/ml) overnight. Images were captured using an Olympus FV1000 Inverted microscope with a Uplan-Apochromat 20×/0.7 Working Distance = 0.65 objective.

### Statistical analysis

Fisher’s exact test can be used to test for dependence by calculating how extreme observed differences in ratios are, given no dependence. We used this test with different kinds of counts in this study. In relation to disorder predictions, we used Fisher’s exact test to investigate whether STRs and the predicted features were dependent. Given no dependence between two features (for instance, between STRs and predicted IDRs), we would not expect a large difference between ratios. Hence, we constructed contingency tables and used the “fisher_exact” function of the Python module “statsmodels” to calculate odds ratios and *P* values from contingency tables (*54*). The odds ratio would be $\frac{A}{B}/\frac{C}{D}$ for a given type of amino acid, where *A* is the count of STR-encoded amino acids in the predicted region, *B* is the count of amino acids not encoded by STRs in the predicted region, *C* is the count of STR-encoded amino acids not in the predicted region, and *D* is the count of amino acids not encoded by STRs and not in the predicted region (see legend dataset S1). Here, we used two-sided Fisher’s exact test, as we were interested in both positive and negative dependence. We used one-sided Fisher’s exact tests to address whether there was dependence between IDRs or predicted IDR binding sites and associations with gene expression (see legend dataset S4).

We used ordinary linear square regression to test whether the environment of *Arabidopsis* accessions could be predicted by allelic variation in coding STRs. The modeling was performed using the “ols” function of the statsmodels Python package. We ran OLS regression with the seven PC axes as a response, separately, and each STR separately. In addition, STRs were modeled as categorical variables, which tests for nonlinear STR effects. For multiallelic STRs (more than two variants), we compared the nonlinear STR effect model with the linear model using the “anova_lm” function of statsmodels and kept the *P* value and *R*^2^ of the best model. Mock STRs were generated by shuffling the real STR genotypes randomly among the accessions, per STR site, and were modeled in the exact same manner as the real STRs. For purposes of correcting for population structure, we used linear mixed models as described in (*9*). Briefly, we used the “qtl.scan” function of the Python package “limix” (v3.0.4; https://github.com/limix/limix), setting *G* as the STR genotypes, *Y* as the environmental PC axes, and *K* as the standardized genetic correlation matrix. Models with and without *G* were then compared, and *P* values were calculated by comparing the likelihood ratios of the two models.

We first used the “rda” function of the R package “vegan” to decompose the standardized correlation matrix into PC axes 1 and 2. We used the vegan function “envfit” (*55*) to treat the seven environmental PC axes as responses in multiple regressions with the PC axes as explanatory variables (environmental variable ~ PC axis 1 + PC axis 2) to produce *P* and *R*^2^ values used to assess the extent of explained variation.

We used the ols function of the statsmodels Python package to run multiple linear regression with the command “smf.ols(‘bestR2 ~ 1 *+* AA + protein_binding + DNA_binding + RNA_binding + in_disorder’, data = data, hasconst = True).fit(),” where bestR2 is the squared correlation (*R*^2^) resulting from the environmental association analysis; *1* is the intercept; *AA* is the repeated amino acid in the tract (as a categorical variable); “protein_binding” is an ordinal variable with values 0, 1, 2, or 3 depending on the extent the three different PPI prediction tools agreed (for instance, 3 if all tools agreed); and “DNA_binding,” “RNA_binding,” and “in_disorder” were treated as binary (0 or 1) variables. We repeated the modeling using the Bayes factor stemming from Bayenv2.0 and the *F*_ST_ value from the *F*_ST_ analysis as response variables.

The luciferase measurements were performed 12 times per construct. We used a Welch *t* test to test whether the differences in mean luciferase outputs were statistically significant.

## Supplementary Materials

### This PDF file includes:

Figs. S1 to S9 Tables S1 to S4 Legends for datasets S1 to S14

### Other Supplementary Material for this manuscript includes the following:

Datasets S1 to S14

## Notes

View/request a protocol for this paper from *Bio-protocol*.
