# Supplementary material for: Adaptive protein evolution through length variation of short tandem repeats in Arabidopsis
Source: Sci Adv. 2023 Mar 22;9(12):eadd6960. doi: 10.1126/sciadv.add6960 (PMC10032594; doi:10.1126/sciadv.add6960)
Supplement: Supplementary file 1 — Figs. S1 to S9 Tables S1 to S4 Legends for datasets S1 to S14 [file sciadv.add6960_sm.pdf]

Science Advances
AAAS

Supplementary Materials for

Adaptive protein evolution through length variation of short tandem repeats in *Arabidopsis*

William B. Reinar *et al.*

Corresponding author: Kjetill S. Jakobsen, k.s.jakobsen@ibv.uio.no; William B. Reinar, w.b.reinar@ibv.uio.no

Sci. Adv. **9**, eadd6960 (2023)
DOI: 10.1126/sciadv.add6960

The PDF file includes:

Figs. S1 to S9
Tables S1 to S4
Legends for datasets S1 to S14

Other Supplementary Material for this manuscript includes the following:

Datasets S1 to S14

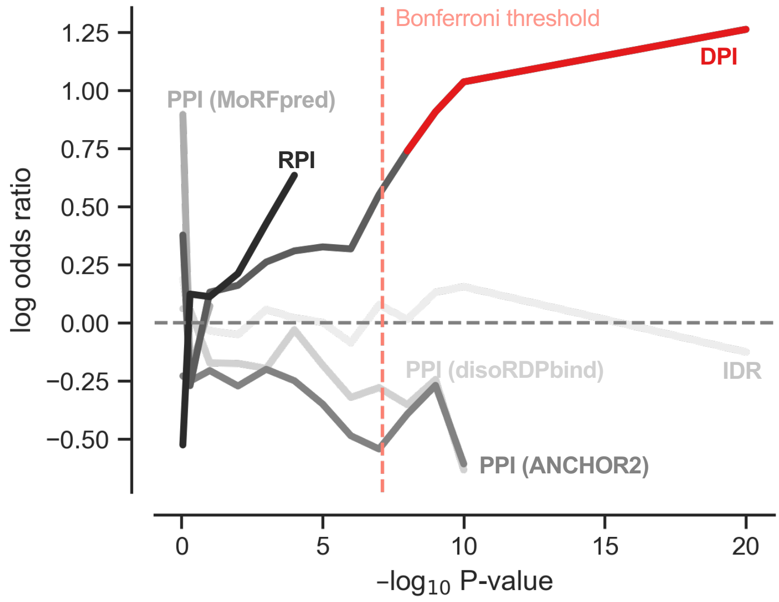

Fig. S1. STR-encoded homopolymers that overlap predicted disordered DNA-binding regions were overrepresented in being associated with autoregulatory gene expression compared to other protein coding STRs. The x-axis shows the $-\log_{10}$ P-value from associations between length variation and gene expression, retrieved from (*9*), and the y-axis shows the degree of enrichment/depletion of the tracts in the different predicted regions. The light red, dashed line indicates the adjusted P-value threshold after adjusting for multiple tests (Bonferroni) in the gene expression analysis. The solid lines denote the log odds ratio resulting from the Fisher's Exact of dependence and is colored in shades of gray if the P-value was above the alpha threshold (0.05), and colored dark red otherwise. See Dataset S4 for the source data.

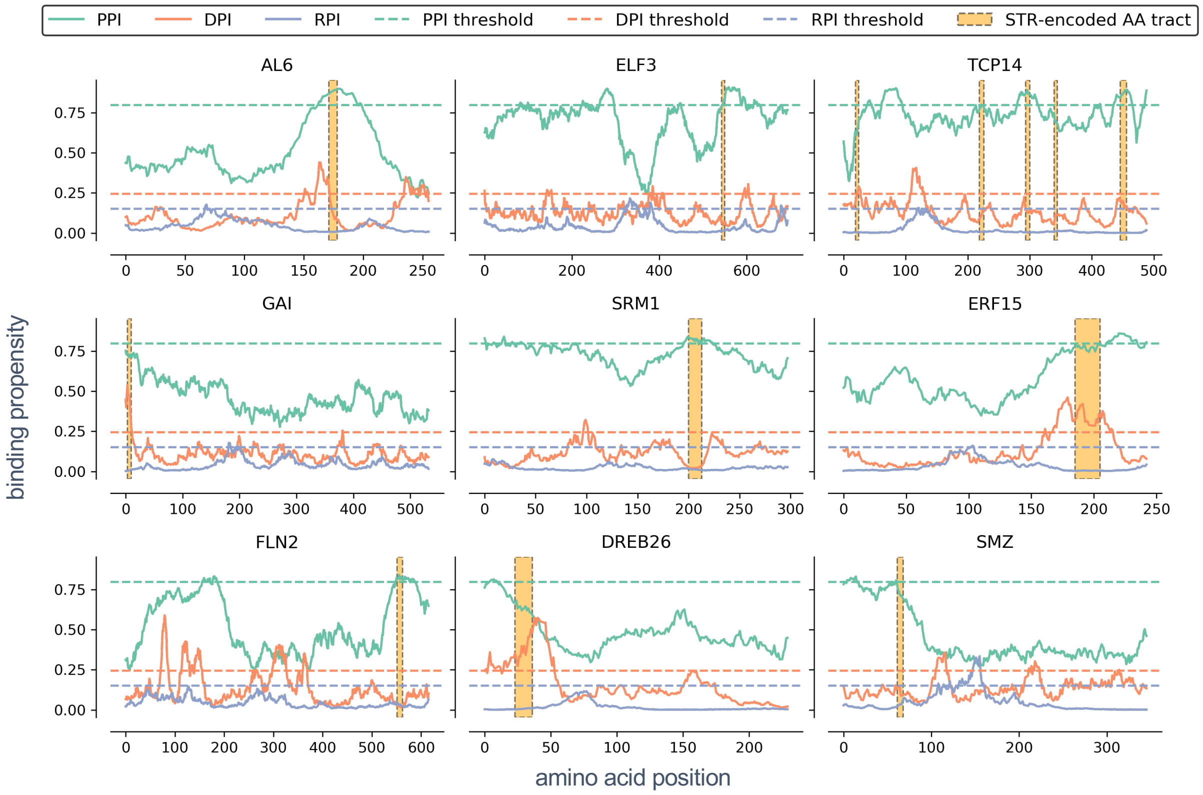

Fig. S2. Examples of disordered interaction region prediction diagrams. The y-axis shows the binding propensity as predicted by DisoRDPbind. The x-axis shows the coordinates of the protein sequences. PPI: protein-protein interaction, DPI: DNA-protein interaction, RPI: RNA-protein interaction. Only predictions from DisoRDPbind are shown. The glutamate tract in ALFIN-LIKE 6 (AL6) influences the activation of its own promoter and the interaction with REALLY INTERESTING NEW GENE 1A (RING1A) (9). Length variation in a glutamine tract in EARLY FLOWERING 3 (ELF3) correlates with thermal responsiveness (5), and overlaps a predicted PPI region. TEOSINTE BRANCHED1, CYCLOIDEA, AND PROLIFERATING CELL FACTOR 14 (TCP14) contain many STR-encoded amino acid tracts, and multiple of these were linked to elevated protein-protein binding propensity. GIBBERELLIC ACID INSENSITIVE (GAI), SALT-RELATED MYB 1 (SRM1), ETHYLENE-RESPONSIVE TRANSCRIPTION FACTOR 15 (ERF15), FRUCTOKINASE-LIKE 2, CHLOROPLASTIC (FLN2), DEHYDRATION-RESPONSE ELEMENT-BINDING PROTEIN 26 (DREB26) and SCHLAFMUTZE all contain amino acid tracts that overlap elevated binding propensities.

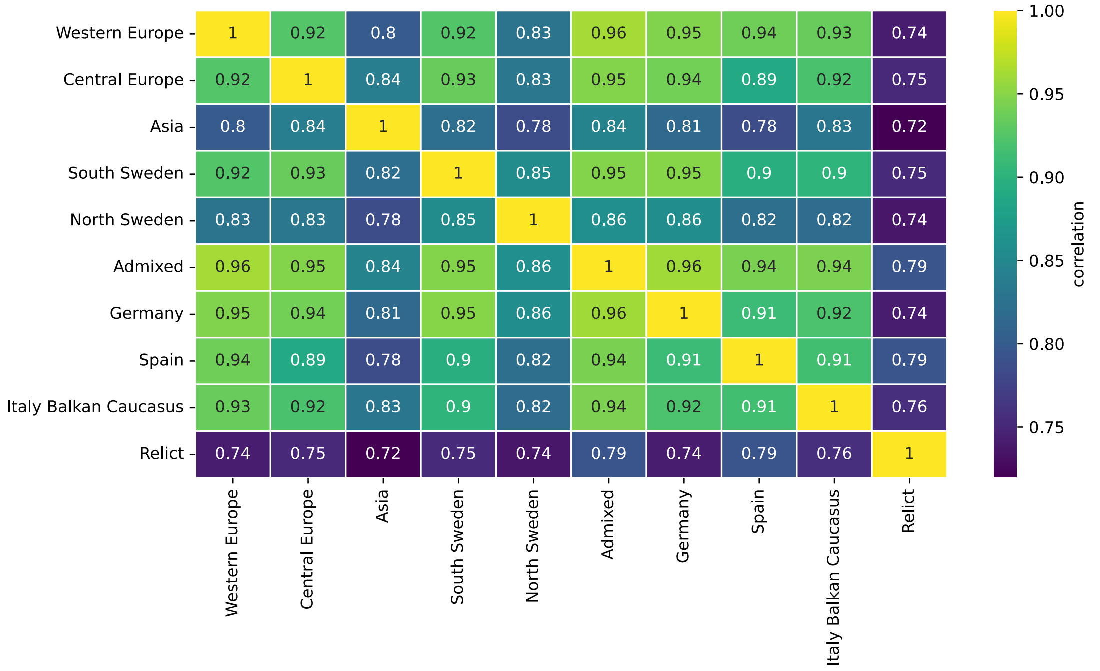

Fig. S3. Correlation matrix of STR-encoded homopolymer variant allele frequencies in *Arabidopsis thaliana* subpopulations/groups. The variance-covariance equivalent of the correlation matrix served as input to Bayenv2.0 to control for genetic similarities.

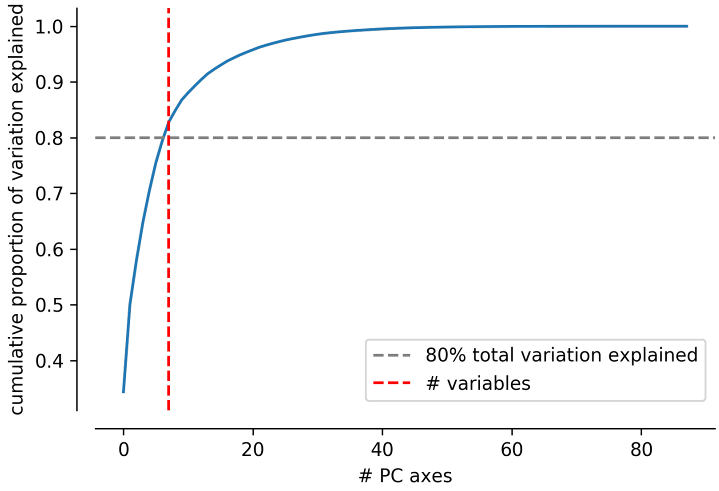

Fig. S4. The cumulative proportion of environmental variation explained by principal component (PC) axes.

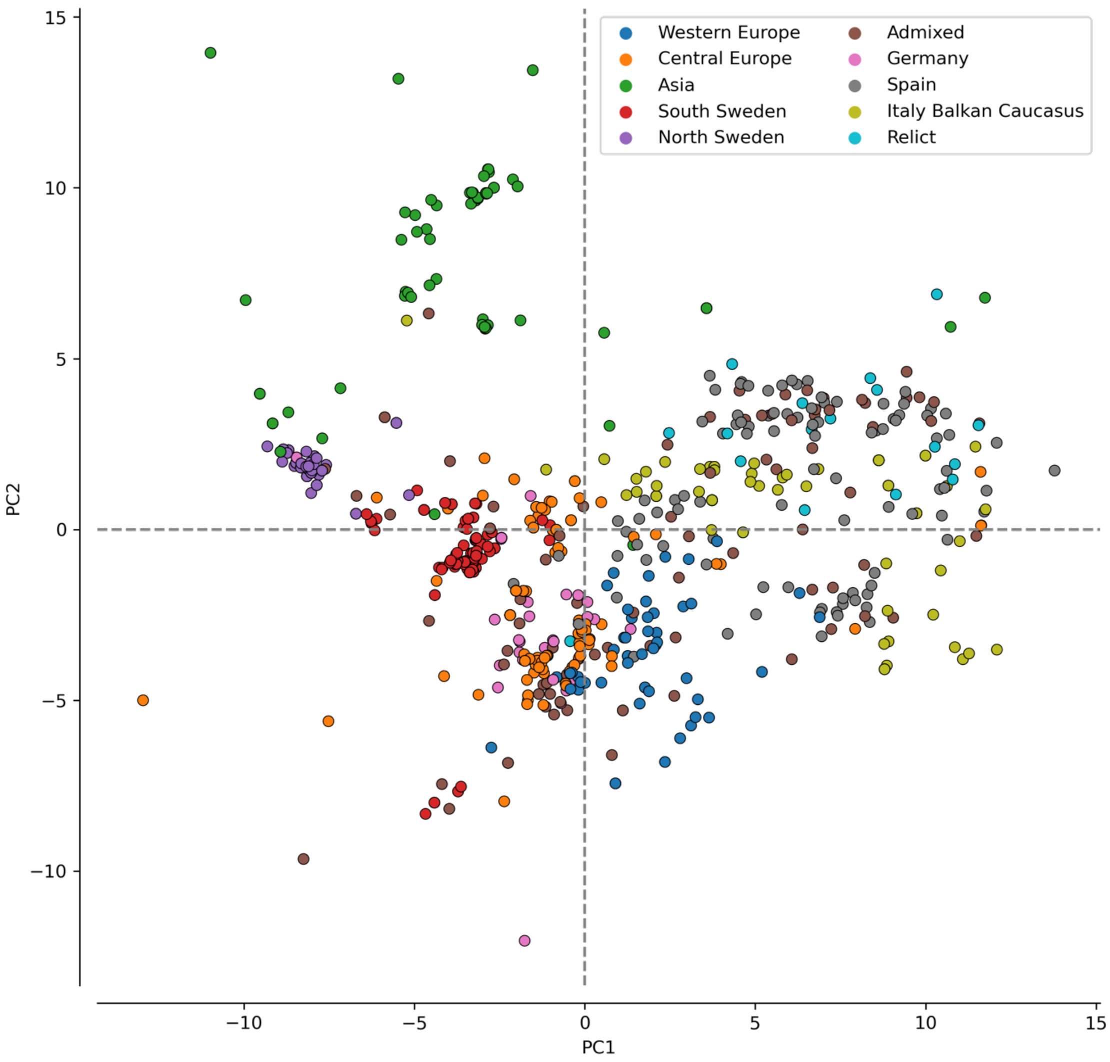

Fig. S5. The position of the 770 *Arabidopsis thaliana* accessions included in this study on principal component (PC) axis 1 and PC axis 2. The first axis captured 34.4% of the variation in the 88 environmental variables (listed in dataset S6), and the second axis captured 15.7% of the variation. Accessions are colored by the groups designated by the 1001 Genomes Consortium (10).

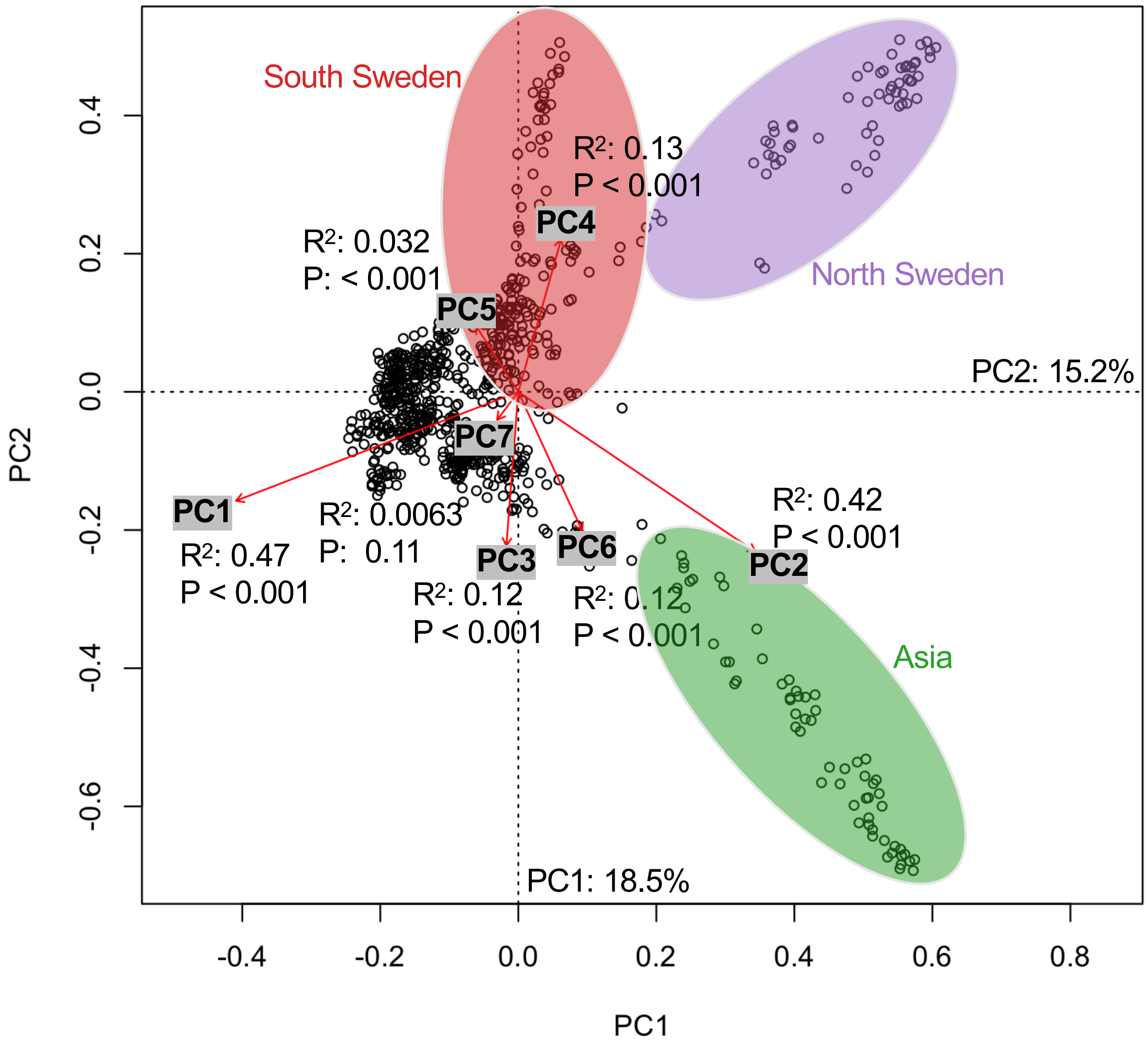

Fig. S6. Putative neutral markers are significantly structured by environmental principal component (PC) axes 1 and 2. PC axis 1 (x-axis) and PC axis 2 (y-axis) represents gradients that best explain the structuring of accessions, based on putative neutral SNPs. Red arrows denote the environmental PC axes projected in the genetic space. Arrows are scaled with R^2, and indicate to what extent the genetic structure can be explained by the environmental PC axes. For clarity, the three most prominent clusters (South Sweden, North Sweden and Asia) are highlighted (a few non-South Sweden accessions are present in the South Sweden cluster).

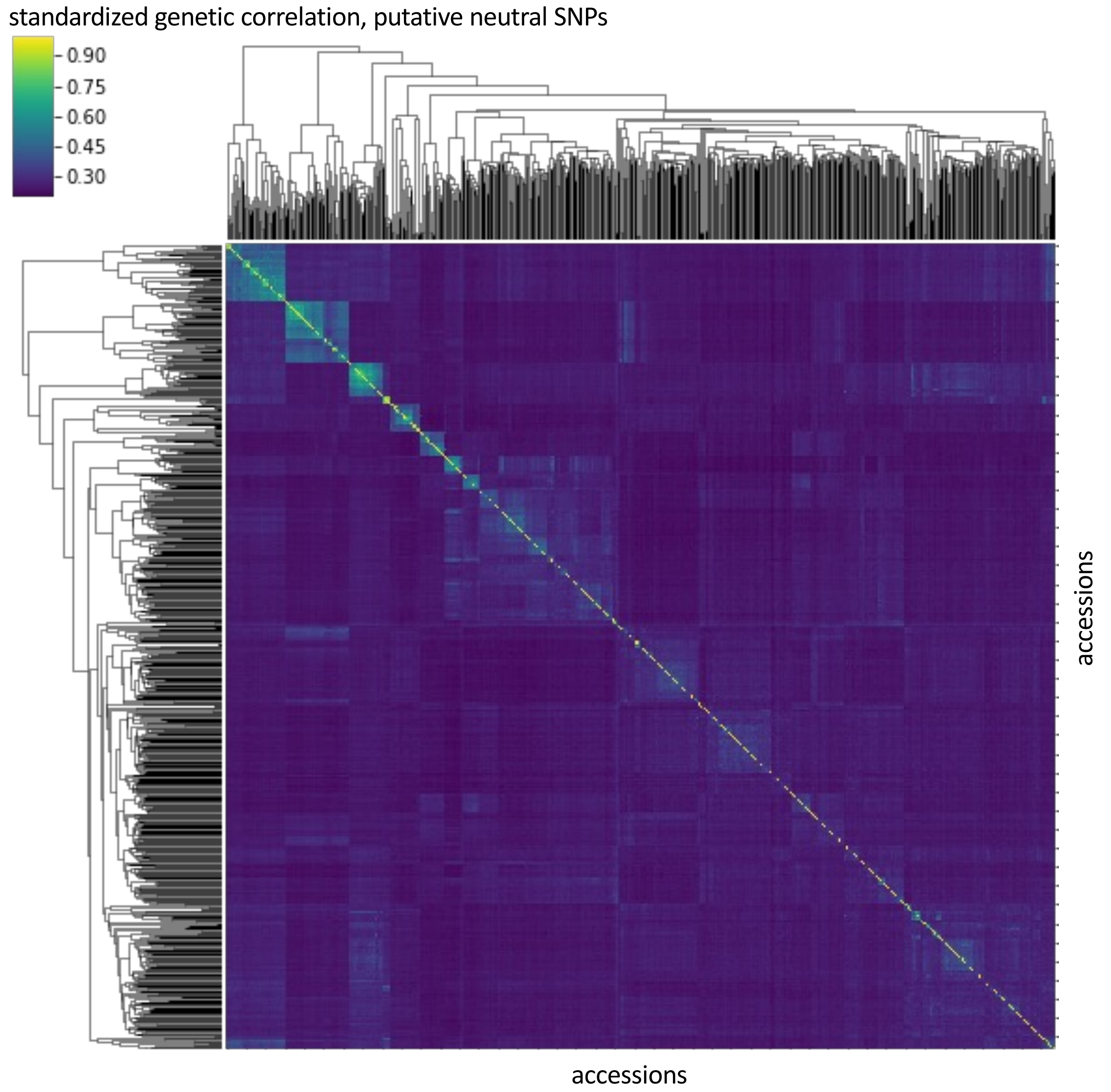

Fig. S7. Standardized pairwise correlation matrix of putative neutral genetic markers. Higher values (from dark blue to yellow) denote more genetically similar pairs of accessions.

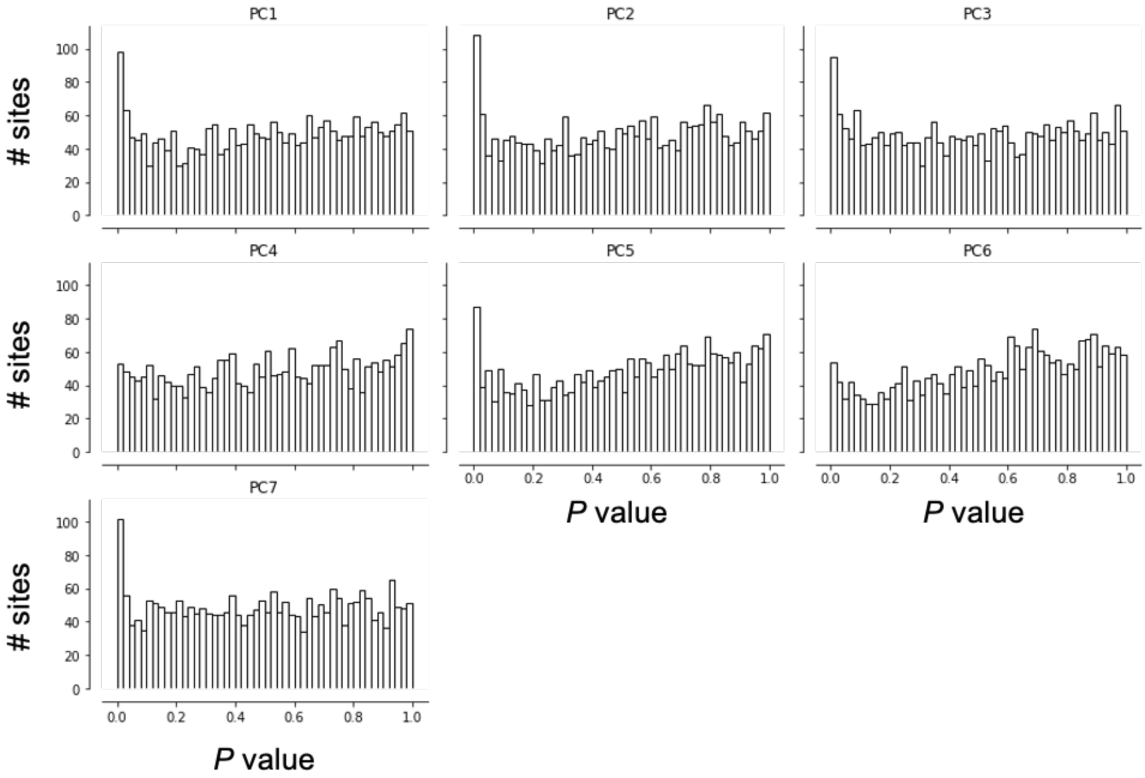

Fig. S8. P-value histograms resulting from environmental association analyses with correction for putative neutral genetic markers.

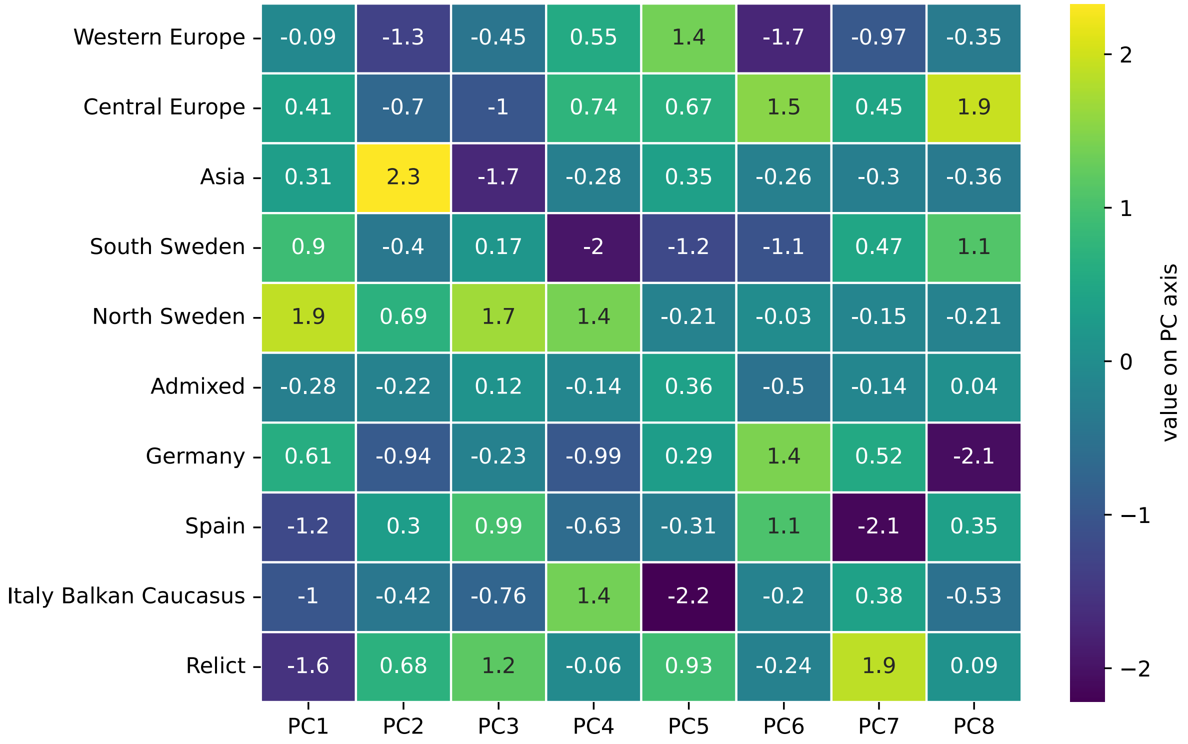

Fig. S9. The mean environmental PC axes values of the ten Arabidopsis thaliana subpopulations/groups. The matrix shows the value of each subpopulation (y-axis) on the

eight (scaled) complex environmental gradients (PC1-8, x-axis), based on the decomposition of the variation in their mean environmental values, serving as environmental parameters in Bayenv2.0.

Table S1. **The number of different STR-encoded amino acid tracts in the *Arabidopsis thaliana* proteome.** Numbers were based on the primary transcript only (ATXXXXXXX.1), to avoid duplicate counts of tracts. Only tracts containing at least four repeated amino acids in the Col-0 reference protein are shown.

repeated amino acid in tract	# tracts
A	58
C	1
D	306
E	492
F	39
G	215
H	119
I	2
K	263
L	38
M	20
N	157
P	207
Q	169
R	19
S	485
T	81
V	9
Y	2

Table S2. Ordinary Least Square regression results of length variation in the second poly-Q tract of TCP14 and environmental variables. Only tests passing the Bonferroni threshold for multiple testing is shown.

linear model, P-value	linear model, R^2	non-linear effect model, P-value	non-linear effect model, R^2	environmental variable
0.11	0.002	6.94×10^{-5}	0.031	WB_GHI
1.58×10^{-6}	0.031	3.81×10^{-5}	0.030	NPP_Spring
0.11	0.003	0.00011	0.030	WB_PVOUT
0.088	0.003	0.00011	0.030	WB_GTI
1.58×10^{-6}	0.031	3.81×10^{-5}	0.030	NPP_Spring
0.00010	0.020	7.96×10^{-5}	0.028	GPCC_Fulldata_Precipitation
0.00010	0.020	7.96×10^{-5}	0.028	GPCC_Fulldata_Precipitation
0.00093	0.014	0.00022	0.025	Net_radiation_summer
2.67×10^{-5}	0.023	0.00064	0.022	CHELSA_BIO7
6.06×10^{-5}	0.023	0.00162	0.021	GPP_Spring
2.81×10^{-5}	0.023	0.001	0.020	longitude
0.00017	0.018	0.0023	0.018	Distance_to_the_coast
0.00015	0.019	0.0034	0.017	CHELSA_BIO4

Table S3. Primers used in this study.

Primer	sequence
TCP14 CDS Attb1	5'GGGGACAAGTTTGTACAAAAAAGCAGGCTCAATGCAAAAGCCAACATCAAGTATC '3
TCP14 CDS Attb2	5'GGGGACCACTTTGTACAAGAAAGCTGGGTTATCTTGCTGATCCTCCTCATCAC '3
ELIP1pro Attb1	5'GGGGACAAGTTTGTACAAAAAAGCAGGCTCAAATCAAACATTGGGATTATCGA '3
ELIP1 pro Attb2	5'GGGGACCACTTTGTACAAGAAAGCTGGGTTTATTAACTTATCAATATCGAAAGGC '3

Table S4. Top negative and positive correlations between individual environmental variables and environmental principal component (PC) axis 1-7. The names of all variables used in the PC analysis are available in dataset S6. The variables were collected in (17) and further descriptions of the variables can be found in that study.

correlation (Pearson's R)	variable	category	PC axis
–0.82	CRU Frost day frequency spring	temperature	1
0.92	CRU Monthly average daily maximum temperature spring	temperature	1
–0.80	FAO NPP based on GPCP	productivity	2
0.71	CHELSA BIO4 – Temperature seasonality	temperature	2
–0.55	FAO Sensitivity of NPP with respect to changes in annual mean precipitation dNPP/dT (dNPPdP)	productivity	3
0.69	CRU Precipitation spring	precipitation	3
–0.51	CHELSA BIO2 – Mean diurnal range	temperature	4
0.51	FAO CRU Precipitation	precipitation	4
–0.33	CHELSA BIO8 – Mean temperature of wettest quarter	temperature	5
0.82	FAO GAEZ Toxicity	edaphic	5
–0.28	CHELSA BIO3 – Isothermality	temperature	6
0.71	FAO GPCC Fulldata Precipitation	precipitation	6
–0.49	CHELSA Minimum temperature spring	temperature	7
0.30	ISRIC WISE Soil carbonate carbon density	edaphic	7

Dataset S1. Results of Fisher's exact test of dependence between amino acids encoded by STRs and STRs in predicted disorder or predicted disordered binding sites.

Dataset S2. Coding STR matrix of diploid short tandem repeat unit counts, 770 accessions.

Dataset S3. Results from running Gene Ontology and protein family (PFAM) enrichment analysis via the STRING database.

Dataset S4. Source data and results of Fisher's exact test of dependence between STRs in predicted disorder or predicted disordered binding sites and being associated with differences in gene expression levels.

Dataset S5. Results of sequencing *TCP14* (Col-0, CS76778) and *TCP14* (Schip-1, CS77239).

Dataset S6. Names of the 88 numerical environmental variables with complete data on the 770 *Arabidopsis thaliana* accessions analyzed in this study.

Dataset S7. The 770 *Arabidopsis thaliana* accessions' values on the environmental PC axes 1-7.

Dataset S8. Results of the environmental association (Ordinary Least-Squares) analysis.

Dataset S9. Results of the environmental association analysis (Ordinary Least-Squares) using mock STR genotypes.

Dataset S10. Results of the environmental association analysis (limix) with population structure correction.

Dataset S11. Results of the environmental association analysis (Bayenv2.0).

Dataset S12. Results of the F_{ST}-analysis.

Dataset S13. Output of OLS models with R2/Bayes Factor/FST as responses and amino acids/disorder predictions as explanatory variables.

Dataset S14. Relative light unit measurements, source data Fig. 2C.
