# Supplementary material for: Adaptive protein evolution through length variation of short tandem repeats in Arabidopsis
Source: Sci Adv. 2023 Mar 22;9(12):eadd6960. doi: 10.1126/sciadv.add6960 (PMC10032594; doi:10.1126/sciadv.add6960)
Supplement: Supplementary file 2 — Datasets S1 to S14 [file sciadv.add6960_datasets_s1_to_s14.zip › add6960_Dataset_S5.rtf]

>TCP14_Col0CCCCCCCAATTTTTTCTATGCCACCCCCATTCCCATTCCAAAAGTCCCACCGCCGGCAACCCAAAAAGGGTTATTCCAAACCCAAGGGGAATCTGCGGTGGTGGCAGCTAAAAAGCCACCGTTGAAACGAGCGTCGACGAAAGACCGACACCCGAAAGTAGACGGAAGAGGGAGGAGAATAAGGATGCCGGCGTTATGTGCAGCTAGGGTTTTTCAGCTAACGCGAGAGCTAGGTCATAAATCCGACGGTGAGACAATAGAGTGGCTTCTTCAACAAGCTGAACCATCTGTAATCGCCGCCACCGGAACCGGAACAATCCCGGCGAATTTCACTTCTTTAAACATCTCTCTCCGTTCTTCAGGCTCTTCCATGTCTCTTCCTTCTCATTTCCGCTCCGCCGCTTCCACTTTTAGCCCTAATAACATATTTTCTCCGGCGATGCTTCAACAACAACAACAACAACAACGTGGTGGTGGTGTTGGGTTTCATCATCCCCATCTACAGGGACGTGCACCTACGTCGTCTTTGTTTCCTGGTATTGATAACTTCACACCAACGACGTCGTTTTTGAACTTTCATAATCCAACAAAGCAAGAAGGAGATCAAGATTCTGAAGAGTTAAACTCGGAGAAGAAAAGAAGAATCCAAACGACGTCGGATTTGCATCAACAACAACAACAACACCAACATGATCAAATCGGAGGATATACACTTCAATCTAGCAACAGTGGATCTACAGCCACCGCAGCCGCCGCGCAACAAATACCGGGAAATTTCTGGATGGTTGCGGCGGCTGCGGCTGCAGGTGGTGGTGGTGGTAATAACAACCAAACAGGTGGTCTTATGACAGCTTCTATTGGTACTGGTGGCGGCGGTGGAGAGCCTGTTTGGACGTTTCCTTCCATTAACACGGCAGCGGCAGCGTTATATAGAAGTGGCGTTTCGGGCGTTCCAAGCGGCGCGGTTTCTAGCGGTTTACATTTTATGAATTTCGCAGCGCCAATGGCATTTCTTACTGGACAACAACAGCTAGCAACAACTAGTAATCATGAGATTAATGAAGATAGTAATAATAATGAAGGAGGAAGAAGTGACGGTGGTGGTGATCATCATAATACACAGAGACATCATCATCATCAACAACAACATCATCATAATATTCTCTCCGGCTTGAACCAGTACGGACGGCAAGTTTCCGGCGACTCTCAAGCTAGTGGATCACTTGGAGGTGGTGATGAGGAGGATCAGCAAGATAACCCAGCTTTCTTGTACAAAGTTGGCATTATAAGAAAGCATTGCTTATCAATGGTTGCAACGAACAGGTCACTATCAGTCAAAATAAAATCATTATTGCCATCCAGCTGATTCCCCTATGGTTCCCC>TCP14_CS77239AGGAATTTGCGGTGGTGGCAGTAAAAAGCCCCCCGTGAAACGAGGGTTGACGAAAGACCGACACCCGAAAGTAGGCGGAAGAGGGAGGAGAATAAGGATGCCGGCGTTATGTGCAGCTAGGGTTTTTCAGCTAACGCGAGAGCTAGGTCATAAATCCGACGGTGAGACAATAGAGTGGTTCTTCAACAAGCTGAACCATCTGTAATCGCCGCCACCGGAACCGGAACAATCCCGGCGAATTTCACTTCTTTAAACATCTCTCTCCGTTCTTCAGGCTCTTCCATGTCTCTTCCTTCTCATTTCCGCTCCGCCGCTTCCACTTTTAGCCCTAATAACATATTTTCTCCGGCGATGCTTCAACAACAACAACGTGGTGGTGGTGTTGGGTTTCATCATCCCCATCTACAGGGACGTGCACCTACGTCGTCTTTGTTTCCTGGTATTGATAACTTCACACCAACGACGTCGTTTTTGAATTTTCATAATCCAACAAAGCAAGAAGGAGATCAAGATTCTGAAGAGTTAAACTCGGAGAAGAAAAGAAGAATCCAAACGACGTCGGATTTGCATCAACAACAACAACAACACCAACATGATCAAATCGGAGGATATACACTTCAATCTAGCAACAGTGGATCTACAGCCACCGCAGCCGCCGCGCAACAAATACCGGGAAATTTCTGGATGGTTGCGGCGGCTGCGGCTGCAGGTGGTGGTGGTGGTAATAACAACCAAACAGGTGGTCTTATGACAGCTTCTATTGGTACTGGTGGCGGCGGTGGAGAGCCTGTTTGGACGTTTCCTTCCATTAACACGGCAGCGGCAGCGTTATATAGAAGTGGCGTTTCGGGCGTTCCAAGCGGCGCGGTTTCTAGCGGTTTACATTTTATGAATTTCGCAGCGCCAATGGCATTTCTTACTGGACAACAACAGCTAGCAACAACTAGTAATCATGAGATTAATGAAGATAGTAATAATAATGAAGGAGGAAGAAGTGACGGTGGTGGTGATCATCATAATACACAGAGACATCATCATCATCAACAACAACAACAACAACATCATCATAATATTCTCTCCGGCTTGAACCAGTACGGACGGCAAGTTTCCGGCGACTCTCAAGCTAGTGGATCACTTGGAGGTGGTGATGAGGAGGATCAGCAAGATAACCCAGCTTTCTTGTACAAAGTTGGCATTATAAGAAAGCATTGCTTATCAAGGTGTTGCAACGAACAGGTCACTATCAGTCAAAATAAAATCATTATTGCCATCCAGCTGATTCCCCTTGGTTCCCT
