# Supplementary material for: Adaptive protein evolution through length variation of short tandem repeats in Arabidopsis
Source: Sci Adv. 2023 Mar 22;9(12):eadd6960. doi: 10.1126/sciadv.add6960 (PMC10032594; doi:10.1126/sciadv.add6960)
Supplement: Supplementary file 2 — Datasets S1 to S14 [file sciadv.add6960_datasets_s1_to_s14.zip › add6960_Dataset_S6.rtf]

NO2 SpringNO2 SummerO3 SpringO3 SummerUV index springUV index summerSolar insolation springSolar insolation summerNet radiation springNet radiation summerWATER EQUIVALENT ANOMALY springWATER EQUIVALENT ANOMALY summerPrecipitable water springPrecipitable water summerNDVI SpringNDVI SummerCHELSA Tmin SpringCHELSA Tmin springCHELSA Tmax springCHELSA Tmax summerCHELSA Tmean springCHELSA Tmean summerCHELSA Interann TempCHELSA Pre springCHELSA Pre summerCHELSA Interann preCHELSA BIO1CHELSA BIO2CHELSA BIO3CHELSA BIO4CHELSA BIO5CHELSA BIO6CHELSA BIO7CHELSA BIO8CHELSA BIO9CHELSA BIO10CHELSA BIO11CHELSA BIO12CHELSA BIO13CHELSA BIO14CHELSA BIO15CHELSA BIO16CHELSA BIO17CHELSA BIO18CHELSA BIO19CRU Cld springCRU Cld summerCRU Dtr springCRU Dtr summerCRU Frs springCRU Frs summerCRU Pre springCRU Pre summerCRU Tmp springCRU Tmp summerCRU Tmn springCRU Tmn summerCRU Tmx springCRU Tmx summerCRU Vap springCRUVap summerCRU Wet springCRU Wet summerdNPPdPdNPPdTCRU NPPNPP based on GPCPNPP based on GPCP VASClimOGPCC VASClimO PrecipitationCRU PrecipitationCRU TemperatureGriesser Precip GPCCGPCC Fulldata PrecipitationAridity index of De Martonne GPCC FulldataAridity index of De Martonne GPCC VASClimOAridity index of De Martonne CRUISRIC WISE SoilCarbonate Carbon DensISRIC WISE Soil Org Carbon DensISRIC WISE Soil pHISRIC WISE Total AvailWater capGAEZ Nutrient availabilityGAEZ Nutrient retention capacityGAEZ Rooting conditionsGAEZ Oxygen availability to rootsGAEZ Excess saltsGAEZ ToxicityGrowing degree daysDistance to the coast
